# The Occupational Depression Inventory confounds depressive symptoms with their assumed work-related causes

**DOI:** 10.1177/10519815251327311

**Published:** 2025-03-26

**Authors:** Hannes Zacher, Maie Stein

**Affiliations:** Wilhelm Wundt Institute of Psychology, Leipzig University, Leipzig, Germany

**Keywords:** burnout, depression, employment, measures, occupations, work

## Abstract

**Background:**

Scholars have recently raised concerns regarding the validity of widely-used burnout measures, including the Maslach Burnout Inventory (MBI). At the same time, the Occupational Depression Inventory (ODI) has been proposed as an alternative measure of job-related distress.

**Objective:**

The aim of this commentary is to point out that the items of the ODI confound depressive symptoms that may be experienced by workers, such as lack of energy, feelings of worthlessness, and sleep problems, with their assumed work-related causes, particularly high levels of job stressors.

**Methods:**

This commentary uses conceptual and methodological arguments to describe the problematic consequences of confounded measurement in the ODI.

**Results:**

This commentary suggests that, when researchers use the ODI in empirical studies, confounded measurement can lead to artificially inflated associations between measures of job stressors (e.g., work demands, role conflict, job insecurity) and occupational depression. Moreover, it can be unclear whether associations between occupational depression and potential outcomes (e.g., low job satisfaction, turnover intentions) are caused by depressive symptoms, job stressors, or both.

**Conclusions:**

It is recommended that researchers assess job stressors and workers’ depressive symptoms separately, ideally using multiple sources and time lags to avoid inflated associations between constructs.

Scholars have recently raised concerns regarding the construct validity of widely-used burnout measures, including the Maslach Burnout Inventory (MBI)^1^ and the Oldenburg Burnout Inventory (OLBI),^2^ which assess workers’ experiences of emotional exhaustion, depersonalization/cynicism, and personal inefficacy.^3–5^ We largely agree with these criticisms; for example, it is unclear how the three components can be combined into an overarching burnout construct. As a way forward, Bianchi and colleagues^3^ argue that there are “robust alternatives to the MBI and the OLBI … available” (p. 2), advocating their Occupational Depression Inventory (ODI), which “may help us address job-related distress more effectively” (p. 2). In this commentary, we point out that the ODI is likewise problematic in terms of construct validity. Specifically, the items of the ODI confound key symptoms of depression, such as lack of energy and feelings of worthlessness, with attributions of their work-related causes.

Bianchi and colleagues developed the ODI as a measure “to quantify the severity of work-attributed depressive symptoms and establish provisional diagnoses of job-ascribed depression” (p. 1).^6^ The ODI includes nine items that measure depressive symptoms in the domains of anhedonia, depressed mood, fatigue/loss of energy, appetite alterations, feelings of worthlessness, cognitive impairment, psychomotor alterations, and suicidal ideation. Consistent with general measures of depression, respondents are asked to indicate how often they have experienced the problems mentioned in the items over the past two weeks (4-point scale, including 0 = *never or almost never*, 1 = *a few days only*, 2 = *more than half the days*, 3 = *nearly every day*). The psychometric properties of the ODI have been examined using large worker samples in Australia, France, New Zealand, Switzerland, and the United States.^6,7^ Studies showed that the 9-item ODI scale exhibits strong reliability and a unidimensional structure. For example, an article reported Cronbach's α and McDonald's ω values of .92 and higher, factor loadings ranging from .76 to .90, and item-level explained common variance indices of .80 or higher, suggesting that all of the items contributed homogeneously to the unidimensionality of the ODI measure.^6^ A study with over 12,000 participants further demonstrated measurement invariance of the ODI across 14 countries.^8^ Moreover, findings indicate that the ODI correlated negatively with job satisfaction and general health, and positively with turnover intentions.^6^

However, both the ODI's instructions and items confound depressive symptoms with their assumed work-related causes. The instruction of the ODI reads (italics added): “The following statements concern *the impact your work could have had on you*,” followed by an example item “I felt anxious *because of my job*”.^6^ While this approach may align with the ODI's aim to quantify the severity of work-attributed depressive symptoms, the confounding of cause and effect is problematic from a construct validity perspective. For example, associations between job stressors and occupational depression in empirical studies are likely to be inflated because the assumed causes (i.e., job stressors) are also part of the measurement of the assumed consequences (i.e., occupational depression). This confounding also restricts the scope of investigation to depressive symptoms that workers explicitly attribute to their job, thereby neglecting depressive symptoms that are influenced by job stressors but not linked to them in workers’ perception. Moreover, it is unclear whether associations between occupational depression and potential outcomes (e.g., low job satisfaction, turnover intentions) are caused by depressive symptoms, job stressors, or both.

The item scoring also raises concerns regarding construct validity. Participants are instructed as follows: “If you did NOT feel anxious because of your job, select 0. If you felt anxious for reasons that you consider UNCONNECTED TO YOUR JOB …, select 0 as well. If you felt anxious but don’t know why, again select 0. If it is clear for you that YOUR JOB caused you to feel anxious, select 1, 2 or 3 to indicate how often that happened.” For example, a response of “0” could indicate that people either did not feel anxious in the past two weeks or that they felt anxious for reasons that were – at least in their perception – unrelated to their job.

In designing the items, the scale authors adapted the diagnostic criteria for major depression according to the Diagnostic and Statistical Manual of Mental Disorders (DSM-5)^9^ to specifically refer to one's work/job (italics added): (1) “*My work was so stressful that* I could not enjoy the things that I usually like doing,” (2) “I felt depressed *because of my job*,” (3) “*The stress of my job caused me to* have sleep problems …,” (4) “I felt exhausted *because of my work*,” (5) “I felt my appetite was disturbed *because of the stress of my job* …,” (6) “*My experience at work made me* feel like a failure,” (7) “*My job stressed me so much that* I had trouble focusing on what I was doing (e.g., reading a newspaper article) or thinking clearly (e.g., to make decisions),” (8) “*As a result of job stress,* I felt restless, or the opposite, noticeably slowed down—for example, in the way I moved or spoke,” and (9) “I thought that I’d rather be dead than continue in this job.” A critical observation regarding construct validity is that only items 1, 3, 5, 7, and 8 explicitly refer to (high levels of) job stress/stressors, whereas items 2, 4, and 6 broadly refer to “my job,” “my work,” and “my experience at work” as causes of the respective symptoms. Importantly, however, the inclusion of negative outcomes (e.g., feeling exhausted) in these items implies that people's work experience is aversive.

Item 9 differs from the other eight items in that it does not explicitly attribute suicidal thoughts to the respondent's job. However, its content validity is problematic because respondents are likely to interpret this item metaphorically, reflecting their overall attitude toward their job rather than an indicator of occupational depression.

Our commentary is based on conceptual and methodological arguments regarding the problem of confounded measurement and its potential consequences for construct and criterion-related validity. We are neither questioning the reliability and unidimensionality of the ODI, nor are we suggesting that previous research using the ODI is methodologically flawed. Moreover, the ODI is certainly not the only confounded measure in the work psychology literature; confounded measurement is a pervasive problem. For instance, a measure of employee silence confounds motives (e.g., fear) with the act of being silent at work^10^ and measures designed to assess transformational leadership confound leader behavior with its assumed positive effects on followers.^11^ Moving forward, researchers should refrain from using confounded measures such as the ODI in studies that aim to examine the potential causes and consequences of workers’ mental health. Instead, they should assess workers’ context-free depressive symptoms using self-reports and, separately, measure job stressors using different sources (e.g., supervisors, co-workers, observational, and archival data). For example, the Patient Health Questionnaire (PHQ-9) could be used to assess workers’ depression severity,^12^ and the Copenhagen Psychosocial Questionnaire (COPSOQ) could be used to measure job stressors, such as work demands, role conflicts, and job insecurity.^13^ When only self-reports are feasible, studies should include time lags between measurements to avoid inflated associations between job stressors and depressive symptoms.
